# Supplementary material for: Chemoresistive Nanosensors Employed to Detect Blood Tumor Markers in Patients Affected by Colorectal Cancer in a One-Year Follow Up
Source: Cancers (Basel). 2023 Mar 16;15(6):1797. doi: 10.3390/cancers15061797 (PMC10046137; doi:10.3390/cancers15061797)
Supplement: Supplementary file 1 [file cancers-15-01797-s001.zip › cancers-2207535-supplementary.pdf]

# Chemoresistive Nanosensors Employed to Detect Blood Tumor Markers in Patients Affected by Colorectal Cancer in a One-Year Follow-Up

**Michele Astolfi [1,2], Giorgio Rispoli [3,*], Gabriele Anania [4], Giulia Zonta [1,2] and Cesare Malagù [1,2]**

[1] Department of Physics and Earth Sciences, University of Ferrara, 44122 Ferrara, Italy
[2] SCENT S.r.l., Via Quadrifoglio 11, 44124 Ferrara, Italy
[3] Department of Neuroscience and Rehabilitation, University of Ferrara, 44121 Ferrara, Italy
[4] Department of Medical Sciences, University of Ferrara, 44121 Ferrara, Italy
* Correspondence: rsg@unife.it

## Supplementary Materials

### The device

SCENT B2 comprised three main blocks: a sensing core, a pneumatic circuit, and an electronic system. The sensing core hosts a combination (array) of four concurrently working nanostructured chemoresistive thick-film sensors, based on different semiconductor materials (here metal-oxides). The pneumatic system conveys environmental air at a constant pressure to the sensors, by means of an electronic pump and two flowmeters. Since the sensors are very sensitive to air temperature and humidity, the air flux is stabilized in humidity by a carbon filter and purified from pollutants by a 0.2-micron filter (Figure S1A); the room is maintained at constant temperature of about 21-24 °C [27-30]. The airflow, thanks to a three-way valve, can be directed (by means of two flowmeters) toward the sensing core to set up the baseline (sensor response to environmental air only), or toward the sample box (Figure S2A). In the latter path the airflow gathers the sample (poured in a Teflon container, Figure S2B) emissions accumulated in the sample box headspace, before reaching the sensors. All flowmeters are manually regulated to have a flow-rate through each channel of 0.2 litres per minute (Figure S1A and S2A).

The electronic system consists in five printed circuit boards: the first one is the main board, hosting the device power supply (for the air pump, the sensor boards, the microcontroller, etc.) and an eight-channel i2c multiplexer (TCA9548A) coupling the microcontroller with the four sensor boards (one for each sensor; Figure S2C). The latter host the electronics controlling the sensor heating, its feeding voltage (i.e. $V_i$ in Figure S3), and the 16-bits, analog-to-digital converter (ADS1115). This converter returns one voltage value per sensor every 10 seconds, which is computed as the average value of the last one second of acquisition (at 10 Hz sampling rate). The averaged samples of the four sensors are plotted together in real time by the device management software on the touch-screen of an external computer (Figure S1B) and stored in a .txt file. The management software of SCENT B2 has been completely rewritten in Python environment instead of the SCENT B1 one, which was written in LabView.

The sensor heater temperature is set by a high power (up to 2W) voltage source, driven by a 12-bit DAC (placed on each sensor board; MCP4725) and precisely adjusted by the managing software by measuring the current flowing through the heater.

SCENT B2 electronics was upgraded in respect to SCENT B1 in having more recent components, an updated managing software and communication protocol, an improved signal-to-noise ratio, and a substantial power consumption reduction although with better performances.

# Figures

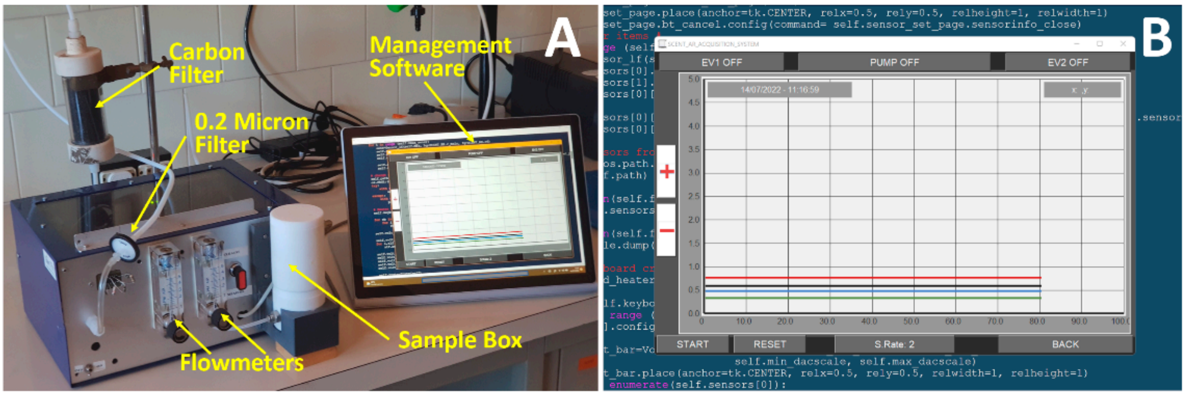


**Figure S1**. The SCENT B2. A, the external view of SCENT B2, where are clearly visible the touch screen display of the external computer, that functions as the user interface as well, the sample box, and the two flowmeters. B, the touch-screen user interface in sensor output mode, graphing the sensor output voltage values $V_0$ (mV).

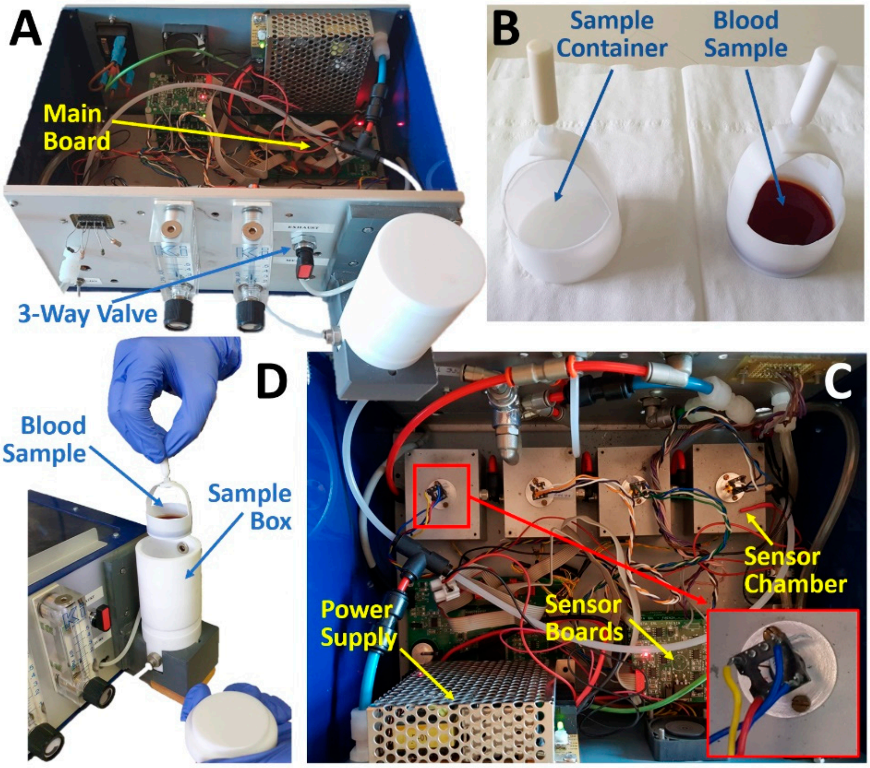


**Figure S2.** Internal details of SCENT B2. A, the device with the top off. B, the reusable Teflon containers: an empty one (left) and a blood filled one (right). C, enlarged view of the internal details, where it can be distinguished the power supply, the four stacked sensor boards and the four sensor chambers (one of which is enlarged in the inset on the left). The 4 mm Teflon tubes form the pneumatic system. D, the Teflon container being inserted in the sample box.

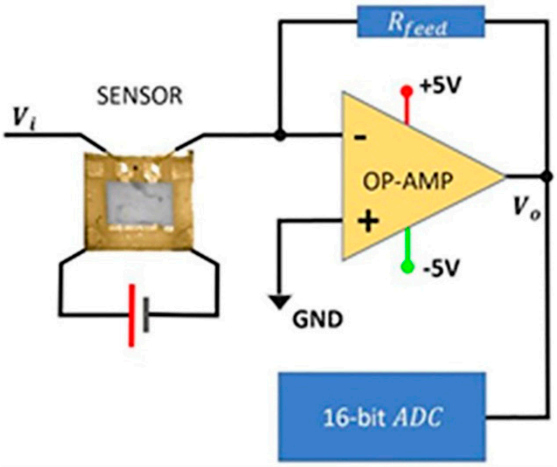


**Figure S3.** Microphotograph of a sensor connected in between the voltage source and the operational amplifier in inverting configuration. The sensor heater contacts are connected to an adjustable voltage source ($V_i$); the output signal $V_0$ is digitized by a 16-bit analog-to-digital converter, handled by the internal microcontroller, and viewed on an external touch screen computer running a custom software written in Python's environment.

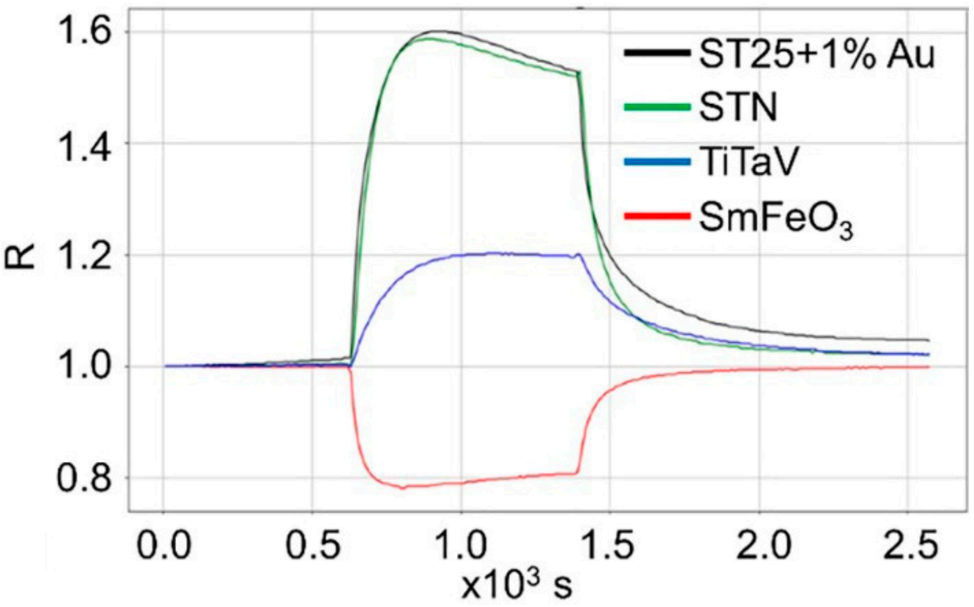


**Figure S4.** Sensor output plot. The response R computed from the voltage in the presence of the gas exhaled by a blood sample and the voltage in the presence of a clean airflow, plotted vs time (Eq. 2).
